# Spermidine inhibits neurodegeneration and delays aging via the PINK1-PDR1-dependent mitophagy pathway in *C. elegans*

**DOI:** 10.18632/aging.103578

**Published:** 2020-09-09

**Authors:** Xin Yang, Mohan Zhang, Yuhua Dai, Yuchao Sun, Yahyah Aman, Yuying Xu, Peilin Yu, Yifan Zheng, Jun Yang, Xinqiang Zhu

**Affiliations:** 1 Central Laboratory of The Fourth Affiliated Hospital, Zhejiang University School of Medicine, Yiwu, China; 2 Department of Toxicology, Zhejiang University School of Medicine, Hangzhou, China; 3 Wenzhou Center for Disease Control and Prevention, Wenzhou, China; 4 Department of Clinical Molecular Biology, University of Oslo, Oslo, Norway; 5 Department of Toxicology, Hangzhou Normal University School of Medicine, Hangzhou, China

**Keywords:** spermidine, neurodegenerative diseases, *caenorhabditis elegans*, mitophagy, aging

## Abstract

Aging is the primary driver of various diseases, including common neurodegenerative diseases such as Alzheimer’s disease (AD) and Parkinson’s disease (PD). Currently there is no cure for AD and PD, and the development of novel drug candidates is demanding. Spermidine is a small anti-aging molecule with elimination of damaged mitochondria via the process of mitophagy identified as a molecular mechanism of action. Here, we show that spermidine inhibits memory loss in AD worms and improves behavioral performance, e.g., locomotor capacity, in a PD worm model, both via the PINK1-PDR1-dependent mitophagy pathway. Additionally, spermidine delays accelerated aging and improves healthspan in the DNA repair-deficient premature aging Werner syndrome (WS) worm model. While possible intertwined interactions between mitophagy/autophagy induction and DNA repair by spermidine are to be determined, our data support further translation of spermidine as a possible therapeutic intervention for such diseases.

## INTRODUCTION

The inevitable biological process of aging is a primary driver of various diseases, such as cardiovascular diseases, cancer, and neurodegenerative diseases. Neurodegenerative diseases, especially Parkinson’s disease (PD) and Alzheimer's disease (AD), exhibit typical age-dependent characteristics, such as genomic instability, telomere attrition, epigenetic alterations, loss of proteostasis, mitochondrial dysfunction, cellular senescence, deregulated nutrient sensing, stem cell exhaustion and altered intercellular communication [1–3]. This point suggests that the factors accelerating aging are also involved in the development of neurodegenerative diseases. Werner syndrome (WS), is a disease characterized by an accelerated aging process. As a classical premature aging disease, etiological exploration of WS can shed light on the mechanisms of normal human aging and facilitate the development of interventional strategies to improve healthspan [4]. Spermidine can ameliorate the damage as a result of oxidative stress in aged mice as well as promotes autophagy via chromatin acetylation. It has been shown to exhibit cross-species anti-aging effects, covering yeast, nematodes, fruit flies, and human cells [5]. Spermidine prolongs lifespan in nematodes, fruit flies, and mice, and suppresses age-induced memory impairment (AMI) in aging fruit flies [6, 7]. The aforementioned benefits of spermidine may be contributed by different mechanisms, but at least spermidine-induced autophagy plays a key role since many of the benefits were dependent on different autophagy pathways [8]. It has been reported that in the *Drosophila* model of PD, spermidine feeding can inhibit the early mortality of human α-synuclein protein heterologous expression. Similarly, administration of spermidine can rescue loss of dopaminergic neurons in PD nematodes expressing α-synuclein and reduce PD-related neurodegeneration, which coincided with induction of autophagy [9].

Mitochondria participate in multiple metabolic pathways (such as oxidative phosphorylation and the tricarboxylic acid cycle), and play a major role in energy production required for normal cell activity [10]. Mitochondrial dysfunction leads to the accumulation of reactive oxygen species (ROS) impairs ATP production and the cell signaling pathways from and to mitochondria, makes neurons susceptible to endogenous and exogenous stress-induced death, thereby accelerating aging and the progression of AD, PD, among others [11–15]. Mitophagy is a sub-type of macro-autophagy that removes damaged or superfluous mitochondria, thereby maintaining mitochondria homeostasis. The PINK1 / PDR-1 pathway is an important mitophagy regulatory pathway in nematodes, while in mammals is PINK1 / Parkin. Under normal circumstances, Parkin is located in the cytoplasm and its E3 ubiquitin ligase activity is inhibited. At physiological condition, PINK1 is transported into the mitochondrial intermembrane space where MPP and PARL cleave the mitochondrial targeting sequence and trans-membrane domain of PINK-1; furthermore, cleaved PINK-1 is degraded by the ubiquitin-proteasome system [16, 17]. Upon mitochondrial damage, an alteration in mitochondrial membrane potential (MMP) prevents the translocation of PINK1, which facilitates anchoring of PINK1 on the outer mitochondrial membrane. Subsequent auto-phosphorylation of PINK1 leads to its activation and translocation of cytosolic Parkin to the mitochondrial membrane. PINK1 in turn, phosphorylates and activates Parkin, an E3 ubiquitin ligase, which conjugates ubiquitin onto various OMM proteins, such as voltage dependent anion channel (VDAC) [18, 19]. Although extensive mechanistic studies of the PINK1 / Parkin (PDR-1) pathway have been performed, the role of PINK1 / Parkin (PDR-1)-dependent mitophagy *in vivo* remains unclear. Multiple studies have found that mitochondrial dysfunction is associated with aging and neurodegenerative diseases [15, 20]. Studies have shown that mitophagy defects appear in postmortem brain tissues of human and mice based on tau and Aβ AD models, as well as in AD patients [21, 22]. Enhancing mitophagy can eliminate AD-related hyperphosphorylation of tau protein in human neuronal cells and reverse memory deficits in the transgenic tau nematodes and mice [21]. Also in PD, studies have found that PD pluripotent stem cell (iPSC) -derived neuronal cells, PD animal models, and brain tissue samples from patients with PD are characterized by mitochondrial dysfunction and its associated oxidative stress and inflammatory response [23–26]. In a series of WRN-deficient cells and WS model nematodes, impaired mitochondrial function and mitophagy were found to mediate accelerated aging of WS. After nicotinamide adenine dinucleotide (NAD^+^) precursor supplementation, mitochondrial function was improved and WS symptoms were improved [27]. In summary, mitochondrial damage is likely to be a common phenomenon underlying many neurodegenerative diseases. As the research on the causality of mitophagy defects in AD, PD and other neurodegenerative diseases continues to progress, it will provide new ideas for the development of drugs inducing mitophagy and promoting the clearance of damaged mitochondria as a strategic therapeutic target.

Dietary spermidine exerts cardioprotective effects through enhanced autophagy, reduces cardiac hypertrophy, improves diastolic function, and can extend mouse lifespan [28]. In addition to autophagy, spermidine also enhances mitophagy in cultured cell lines, including human fibroblasts and cardiomyocytes [28]. Spermidine induces mitophagy mainly by inhibiting mTOR and activating phosphorylation of 5′adenosine monophosphate-activated protein kinase (AMPK) [8, 29], which antagonizes mTORC1 at the functional level and may further facilitate autophagic. In addition, ataxia-telangiectasia mutated protein kinase (ATM)-dependent putative kinase 1 (PINK1)/ Parkin signaling can also be activated by spermidine [30]. However, additional mechanisms between spermidine and mitophagy remain elusive.

Here, we expand spermidine’s scope of potential protective effects during the neurodegenerative diseases and premature aging disease in age-related diseases with WS, PD, and AD disease model nematodes, and investigate the possible underlying mechanisms.

## RESULTS

### Spermidine prolongs lifespan and improves healthspan in the WS *C. elegans*

**Figure 1:**
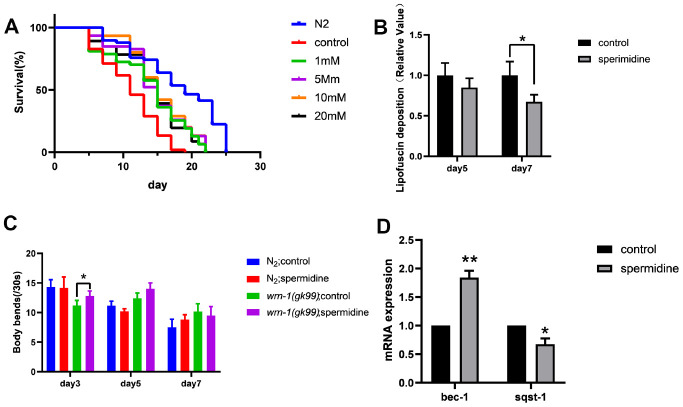
**Effect of spermidine on *wrn-1(gk99)* worms.** (**A**) Effect of different concentrations of spermidine treatment on survival curves of *wrn-1(gk99)* worms, n=83-102. (**B**) Effect of 5mM spermidine treatment on lipofuscin deposition level of *wrn-1(gk99)* worms, n=12-15. (**C**). Effect of 5mM spermidine treatment on locomotor capacity of N2 and *wrn-1(gk99)* worms, n=20. (**D**) Effect of 5mM spermidine treatment on autophagy-related genes *bec-1* and *sqst-1* of *wrn-1(gk99)* worms, n=3. Data are represented as mean± SD, *P < 0.05, **P < 0.01 vs control.

First, in order to evaluate potential of spermidine as a treatment for premature aging, we evaluated the impact on a nematode model of WS namely, *wrn-1 (gk99)*. As expected, the *wrn-1 (gk99)* *C. elegans* exhibited significantly shorter lifespan (median = 11 days) compared to N_2_ wild type (WT) controls (median = 19 days) as previously reported [27]. Spermidine treatment from eggs resulted in an extension of median lifespan, by approximately 36% at the maximal effective dose of 5 mM, in the *wrn-1 (gk99)* worms in a dose-dependent manner compared to vehicle controls. However, doses exceeding 5 mM were no longer beneficial in lifespan extension (Figure 1A). Table 1 summarizes the effects of different concentrations of spermidine on the median survival time of *wrn-1(gk99)*.

**Table 1:** Spermidine dose-average life and median survival of *wrn-1(gk99).*

	**Concentration(mM)**	**Mean lifespan(days)**	**Median survival(days)**
N2(20°C)	0	18.0±0.8	19.0±1.8
	0	11.1±0.5	11.0±0.8
	1	13.8±0.8	15.0±0.7
*wrn-1*(gk99)	5	14.7±0.7	15.0±0.6
	10	14.4±0.6	15.0±0.8
	20	14.3±0.7	15.0±0.7

Subsequently, we evaluated the deposition of intestinal lipofuscin, a characteristic feature that accumulates and increases in an age-dependent manner, which reflect health and the rate of aging in worms [31]. Here we demonstrate, that spermidine (5 mM) prevented the accumulation of intestinal lipofuscin that becomes apparent at the age of adult day 7 in *wrn-1 (gk99)* worms (Figure 1B).

Following observation of spermidine induced increase in lifespan accompanied by reduced age-related accumulation of lipofuscin, in the *wrn-1(gk99)* worms; we evaluated the impact on the locomotor capacity rate of N2 and *wrn-1 (gk99) C. elegans* hermaphrodites moving on an agar surface of a petri plate by counting the number of bends in the anterior body region during a 30 s interval [32]. Supplementation with 5 mM spermidine resulted in increase of locomotor capacity in the *wrn-1 (gk99) C. elegans* at the age of adult day3 and day 5 (Figure 1C).

Spermidine has been shown to stimulate macroautophagy/autophagy [32]. Thus, in order to understand the molecular mechanism underlying the lifespan prolonging and improved healthspan induced by spermidine we evaluated changes in the autophagy machinery. We evaluated the expression levels of autophagy-related genes *bec-1* (homologous to Beclin-1), *sqst-1* (homologous to SQSTM) and *lgg-1* (homologous to LC3). Relative to the vehicle controls, the expression level of *bec-1* was significantly increased (*P*<0.01), meanwhile, the expression of *sqst-1* was significantly reduced (*P* <0.05) following 5 mM spermidine-treatment (Figure 1D). The expression of *lgg-1* was not significantly influenced by spermidine-treatment (Supplementary Figure 1). Gene expression indicated that spermidine induced autophagy may underlie the life-prolonging and improved health in the premature WS nematode model, *wrn-1 (gk99)*.

### Spermidine protects against behavioral deficits and pathological features of PD in *C. elegans* model

**Figure 2:**
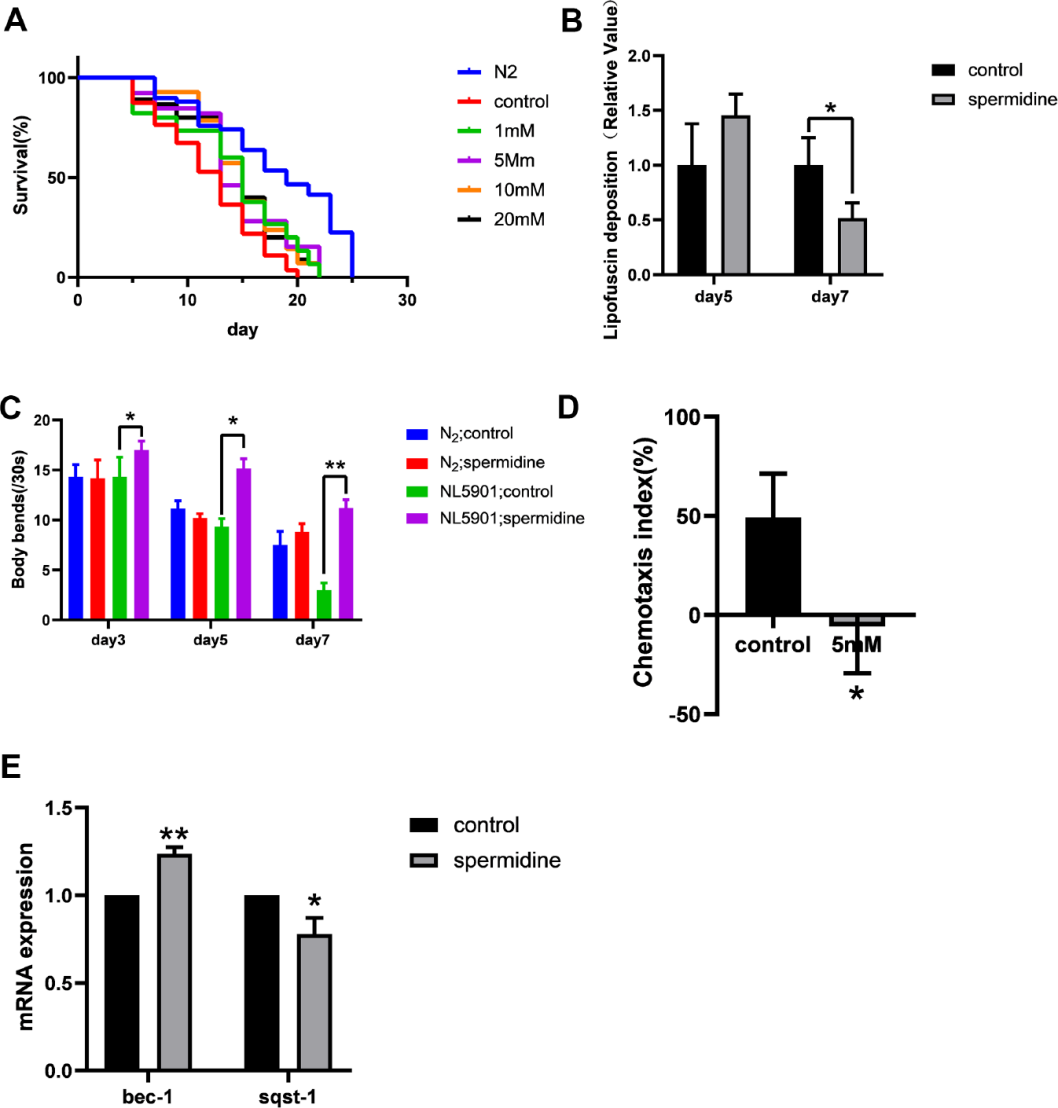
**Effect of spermidine on NL5901 worms.** (**A**) Effect of different concentrations of spermidine treatment on survival curves of NL5901 worms, n=79-100. (**B**) Effect of 5mM spermidine treatment on the level of α-synuclein of NL5901 worms, n=14-18. (**C**) Effect of 5mM spermidine treatment on locomotor capacity of N2 and NL5901 worms, n=20. (**D**) Effect of 5mM spermidine treatment on chemotaxis memory of NL5901 worms, n=3. (**E**) Effect of 5mM spermidine treatment on autophagy-related genes *bec-1* and *sqst-1* of NL5901 worms, n=3. Data are represented as mean± SD, *P < 0.05, **P < 0.01 vs control.

Next, we evaluated the potential of spermidine treatment in a model of neurodegenerative disease, in particular PD. We utilized a well-studied nematode model of PD, the NL5901 strain, in which human α- synuclein fused to YFP is under control of the muscular *unc-54* promoter, transgene pkIs2386 [p_unc-54_::αSYN::YFP] [33]. Muscle expression has been used successfully to model protein-misfolding diseases and to identify modifier genes without considering neuronal effects [33, 34]. The NL5901 strain exhibited significantly shorter lifespan (median = 13 days) to that of the N2 controls (median = 19 days). In order to evaluate the effect of spermidine, we supplemented the NL5901 and N2 with various doses spermidine from eggs. Our data shows that spermidine extends median lifespan of NL5901 model of PD, by approximately 15%, compared to vehicle controls from a dose as low as 1mM (Figure 2A). Table 2 summarizes the effects of spermidine on the median survival time of NL5901.

**Table 2:** Spermidine dose-average life and median survival of NL5901.

	**Concentration(mM)**	**Mean lifespan(days)**	**Median survival(days)**
N2(20°C)	0	18.0±0.8	19.0±1.8
	0	12.1±0.6	13.0±0.8
	1	14.1±0.8	15.0±0.7
NL5901 (20°C)	5	14.4±0.8	15.0±0.6
	10	14.5±0.6	15.0±0.8
	20	14.4±0.7	15.0±0.7

In NL5901 worms, since the fluorescence intensity of α-synuclein is greater than lipofuscin fluorescence, we detected changes of α-synuclein expression. Here we demonstrate, that spermidine (5 mM) prevented the accumulation of α-synuclein that becomes apparent at the age of adult day 7 in NL5901 worms (Figure 2B).

Following observation of spermidine induced increase in lifespan accompanied by reduced accumulation of α-synuclein, in the NL5901 worms; we evaluated the impact on healthspan. First, we quantified the locomotor capacity rate of N2 and NL5901 *C. elegans*. Supplementation with 5 mM spermidine resulted in increase of locomotor capacity in the NL5901 *C. elegans* at the age of adult day 3, day5 and day7 (Figure 2C).

Then, we examined the effect of spermidine supplementation on the cognitive ability of the NL5901 using a chemotaxis-based memory assay. This assay is designed to evaluate the ability of nematodes to learn the association between sodium chloride (NaCl) and food. Under starvation conditions, the nematodes will associate the high NaCl concentration with the starvation signal. After 4h starvation conditioning, in order to escape the A area (this area contains 100Mm NaCl) under starvation, the nematodes would display preference for the B area (this area contains 20mM NaCl). The chemotaxis index (CI) of the vehicle control group was positive, while the CI of the 5 mM spermidine-treated group was negative (Figure 2D). There is a statistical difference between the two groups (*P*<0.05), indicating that NL5901 nematodes are more inclined to move toward the B area with low NaCl concentration after 5mM spermidine treatment, that is, it can better link the starvation signal and the NaCl concentration signal and move more to the B region in order to avoid the high NaCl concentration under starvation.

Similarly, we evaluated the expression levels of autophagy-related genes. Relative to the vehicle controls, the expression levels of *bec-1* was significantly increased (*P*<0.01), meanwhile, the expression of *sqst-1* was reduced (*P*<0.05) following 5 mM spermidine-treatment (Figure 2E). The results showed that spermidine induced autophagy may underlie the life-prolonging and improved health in the PD nematode model, NL5901.

### Spermidine prolongs lifespan and protects against memory loss in the AD *C. elegans*

**Figure 3:**
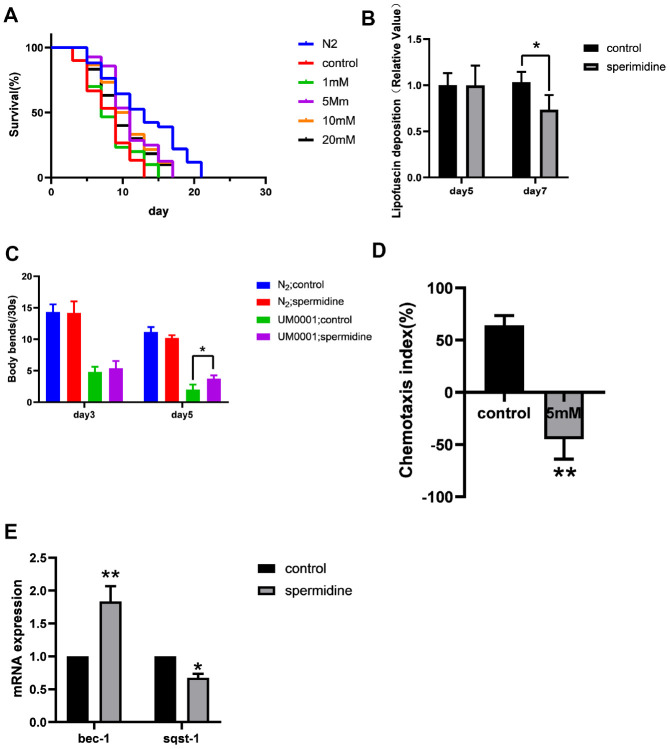
**Effect of spermidine on UM0001 worms.** (**A**) Effect of different concentrations of spermidine treatment on survival curves of UM0001 worms, n=81-98. (**B**) Effect of 5mM spermidine treatment on lipofuscin deposition level of UM0001 worms, n=12-15. (**C**) Effect of 5mM spermidine treatment on locomotor capacity of N2 and UM0001 worms, n=20. (**D**) Effect of 5mM spermidine treatment on chemotaxis memory of UM0001 worms, n=3. (**E**) Effect of 5mM spermidine treatment on autophagy-related genes *bec-1* and *sqst-1* of UM0001 worms, n=3. Data are represented as mean± SD, *P < 0.05, **P < 0.01 vs control.

A nematode model of AD namely, UM0001, in which can co-express human Aβ and tau proteins was used to evaluate the impact of spermidine. As expected, the UM0001 *C. elegans* exhibited significantly shorter lifespan (median = 8 days) compared to N2 wild type (WT) controls (median = 13 days) at 25°C. Spermidine treatment from eggs resulted in an extension of median lifespan, by approximately 37.5% at the maximal effective dose of 5 mM, in the UM0001 worms in a dose-dependent manner compared to vehicle controls. However, doses exceeding 5 mM were no longer beneficial in lifespan extension (Figure 3A). Table 3 summarizes the effects of different concentrations of spermidine on the median survival time of UM0001.

**Table 3:** Spermidine dose-average life and median survival of UM0001.

	**Concentration(mM)**	**Mean lifespan(days)**	**Median survival(days)**
N2(25°C)	0	12.9±0.7	13.0±1.2
	0	8.0±0.4	9.0±0.6
	1	8.4±0.4	7.0±0.6
UM0001(25°C)	5	11.0±0.5	11.0±0.4
	10	10.5±0.5	9.0±0.6
	20	9.9±0.5	9.0±0.5

In addition, we evaluated the deposition of intestinal lipofuscin. Here we demonstrate, that spermidine (5 mM) prevented the accumulation of intestinal lipofuscin that becomes apparent at the age of adult day 7 in UM0001 worms (Figure 2B).

We quantified the locomotor capacity rate of N2 and UM0001 *C. elegans*. Supplementation with 5 mM spermidine resulted in increase of locomotor capacity in the UM0001 *C. elegans* at the age of adult day5 (Figure 3C).

For chemotaxis memory ability, the chemotaxis index (CI) of the vehicle control group was positive, while the CI of the 5 mM spermidine-treated group was negative (Figure 3D). There is a statistical difference between the two groups (*P*<0.01), indicating that UM0001 nematodes are more inclined to move toward the area with low NaCl concentration after 5mM spermidine treatment, that is, it can better link the starvation signal and the NaCl concentration signal.

Again, with respect to the expression levels of autophagy-related genes, relative to the vehicle controls, the expression levels of *bec-1* was significantly increased (*P*<0.01), meanwhile, the expression of *sqst-1* was reduced (*P*<0.05) following 5 mM spermidine-treatment (Figure 3E). Therefore, indicating that spermidine induced autophagy change may underlie the life-prolonging and improved health in the AD nematode model, UM0001.

### Spermidine extends lifespan and improves healthspan via the PINK1-PDR1 pathway

As spermidine has been shown to promote life extension and improve healthspan in models of neurodegeneration and premature aging, we extended our study in order to understand the underlying mechanisms. In particular, due to the association of spermidine with macroautophagy/autophagy [8], which coincides with behavioral and pathological benefits highlighted in the present study; we pursued to explore further in this avenue. Coupled with macroautophagy, recent evidence for contribution of defective mitophagy, a sub-form of autophagy for selective removal of damaged/superfluous mitochondria, to development and progression of AD led this study to shed light on the effect of spermidine on the PINK1-PDR1 axis [21]. For this purpose, we selectively knocked-down the expression of *pink1* and *pdr-1* using RNAi in the models of premature aging (*wrn-1 (gk99)*), Supplementary Figures 2, 3 and neurodegenerative disease (NL5901 and UM0001), and evaluated the effect of spermidine.

**Figure 4:**
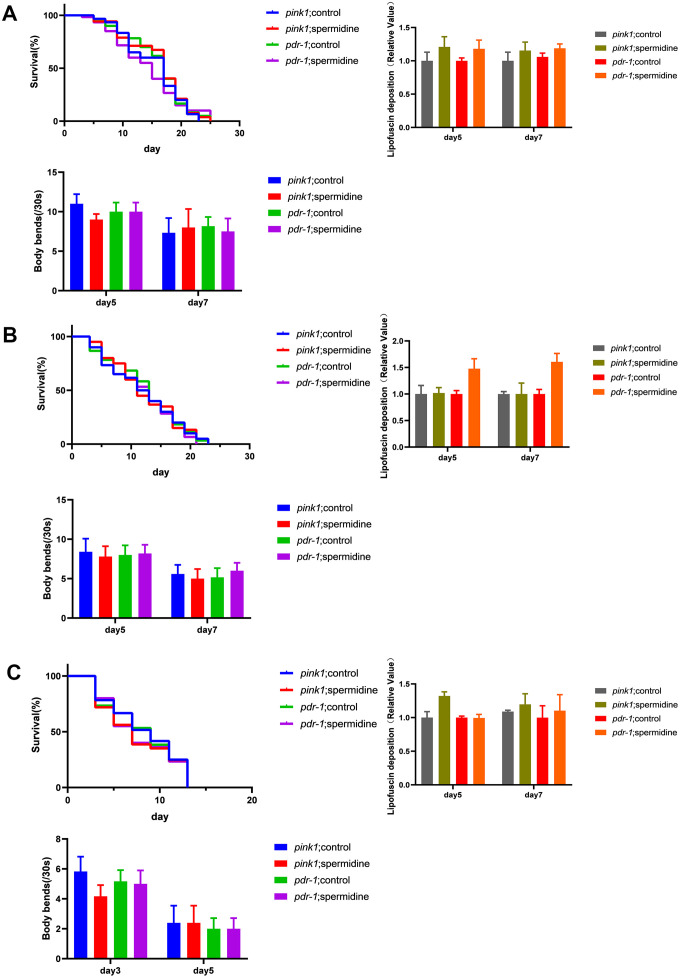
**Effects of spermidine on RNAi knockdown of *pink1* and *pdr-1* nematodes.** (**A**) Effect of spermidine treatment on survival curves(n=85-101), lipofuscin deposition level(n=15), and locomotor capacity of *wrn-1*(gk99) worms(n=20). (**B**) Effect of spermidine treatment on survival curves(n=87-98), α-synuclein level(n=15), and locomotor capacity of NL5901 worms(n=20) (**C**) Effect of spermidine treatment on survival curves(n=92-97), lipofuscin deposition level(n=15), and locomotor capacity of UM0001 worms(n=20). Data are represented as mean± SD, *P < 0.05, **P < 0.01 vs control.

Our results showed that the promoted life extension effects of spermidine disappeared after knocking down the expression of *pink1* and *pdr-1* in WS, PD, AD model nematodes (Figure 4A–4C). Then, we evaluated the deposition of intestinal lipofuscin of *wrn-1 (gk99)* and UM0001, as well as the expression level of α- synuclein of NL5901. Here, we demonstrated that spermidine no longer prevented the accumulation of intestinal lipofuscin in the *wrn-1 (gk99)* and UM0001 nematodes (Figure 4A, 4C) and the expression level of the α- synuclein in NL5901 nematode (Figure 4B). Moreover, we found that the improved locomotor capacity of spermidine treatment disappeared in the three nematodes (Figure 4A–4C).

## DISCUSSION

Spermidine is a naturally occurring polyamine that exhibits diverse biological activities and reduces or delays the onset of age-associated diseases. An age dependent decrease of polyamine levels has been described in many organisms and tissues [35]. Here, we present results for the premature aging and neurodegenerative diseases *C. elegans* models. Through these parallel studies, we sought to identify additional mechanisms by which spermidine may contribute to inhibiting neurodegeneration and delaying aging.

In WS model nematodes, spermidine protected against age-related accumulation of lipofuscin as well as improved the exercise capacity. Collectively, indicating that spermidine delay the aging process of WS model nematodes and improve their health. Compared with the untreated PD model group, spermidine could reduce the expression of α-synuclein protein, and significantly improved the motor ability with injury, and the chemical tropism-mediated learning ability was also improved. It is suggested that spermidine can improve the health level of PD model nematodes, especially greatly improve the motor dysfunction in PD symptoms. This is in line with the results of a study in which spermidine rescued α-synaptic protein-induced dopaminergic neuron loss in PD model nematodes [9]. The most important clinical symptom of AD is severe cognitive impairment. In this study, we confirmed that AD model nematodes inhibited lipofuscin deposition and improved exercise capacity after treatment with spermidine. In terms of chemotaxis memory ability, hunger signals and NaCl signals can be better integrated, and learning ability has been significantly improved in AD model nematodes. This suggests that spermidine may mitigate the cognitive impairment in AD model nematodes, but further confirmation is needed in the future. Spermidine has significantly improved the learning ability of PD and AD nematodes, which is consistent with the results of a study that inhibited memory impairment after supplemented with spermidine in aging fruit flies [6].

Autophagy disorders have become an important pathogenesis of many neurodegenerative diseases. It has been reported that autophagy levels have been reduced in post-mortem brain tissue of patients with AD, PD, and HD, as well as in various cultured cells, fruit fly, nematode, and mouse models of these diseases [36]. Given the phenotypes of the three nematodes after spermidine treatment, we observed that the expression levels of autophagy-related genes were differently changed after spermidine treatment. PINK1 and Parkin (PDR-1 in *C. elegans*) are important pathways for mitophagy and have been shown to play important roles in improving mitochondrial function, so we next investigated the relationship between the protective effect of spermidine and mitophagy. After RNA interference was performed on the PINK-1 and PDR-1 of the three nematodes, and then treated with spermidine, we found that the protective effect of spermidine disappeared, indicating that the function of spermidine achieved is through the pink1 / pdr-1 pathway, but the causal relationship of mitophagy in the protection of spermidine remains to be further studied.

Our experiments obtained positive results on the age-related diseases nematode model, however the use and effects of spermidine in the human is still controversial. For instance, studies show the mechanisms through which oral spermidine intake can mediate systemic effects on blood metabolites and proteins remain to be elucidated. [8]. Given this situation, it still needs to be further verified on spermidine with mammalian models, and finally be confirmed by population studies.

In summary, the herein presented data show that in the *C. elegans* model, spermidine can greatly delay aging, enhance mitophagy levels, ameliorate the symptoms of neurodegenerative and premature aging diseases, which is linked to the PINK1-PDR1-dependent mitophagy pathway. The results of our study provide a novel therapeutic strategy to combat age-related diseases in the future.

## MATERIALS AND METHODS

### *C. elegans*s strains and genetics

Standard *C. elegans* strain maintenance procedures were followed [37]. The following strains were used in this study. N_2_: wild-type. The nematode model of WS *wrn-1* (gk99): it has a deletion mutation of 196 bp, resulting in complete deletion of WRN-1 protein [30, 31]. NL5901 (Punc-54 :: human α-synuclein :: YFP + unc-119):The nematode model of PD. The NL5901 strains were generously provided by Wei Zou (Translational Medicine Research Institute of Zhejiang University). UM0001:The nematode model of AD, obtained by crossing two lines of Aβ1-42 (CL2355) and TAU (BR5270), which can co-express human Aβ and tau proteins [28].

### Culture and spermidine treatment of *C. elegans*

The N_2_, *wrn-1 (gk99)*, NL5901 nematodes were kept on Nematode Growth Medium (NGM) plates at 20° C, the UM0001 was kept at 16° C to L4, then transferred to 25° C. Spermidine was purchased from Aladdin (Shanghai, China). The subsequent treatment of synchronized worm eggs with spermidine (stock, 200 mM in M9 buffer solution) and M9 buffer solution as control. Transferring the nematodes onto a new plate containing spermidine every two days in order to maintain spermidine concentration.

### *C. elegans* lifespan assay

Lifespan analyses were performed on the synchronized eggs. The *wrn-1(gk99)* and NL5901 worms treated with various concentrations of spermidine extracts at 20°C. The survival and dead worms were counted every two day until all nematodes died by determining their movement and pharyngeal pumping. Approximately 100 worms were scored in each experiment.

### *C. elegans* intestinal lipofuscin deposition analysis

The three kinds of nematodes were cultured and treated as described above. The intestinal lipofuscin deposition was measured as described previously with modifications [38]. The autofluorescence of intestinal lipofuscin was measured at day-5 and day-7 adulthood using an epifluorescence microscope (Leica, Wetzlar, Germany) with excitation/emission wavelength of 350 ± 25/460 ± 25 nm. The relative fluorescence intensity was quantified by Image-Pro Plus software (Media Cybernetics, MD, USA). Approximately 20 worms were examined in each experiment.

### Analyses of α-synuclein aggregation

The α-synuclein expressing worms, NL5901, was cultured and treated as described above. The YFP intensity of α-synuclein in the control and treated worms at day-5 and day-7 adulthood were measured by a fluorescence microscope (BX53; Olympus Corp., Tokyo, Japan) and quantified using the Image-Pro Plus software. Approximately 20 worms were examined in each experiment.

### *C. elegans*s locomotion assay

Locomotion was measured on solid media in this study. Briefly, to measure body bending, day-3 and day-5 adulthood nematodes were transferred into 1mL M9 buffer, which was used for washing and maintaining the worms for a short time. Then worms were transferred to NGM plates without bacteria and adjusted for 1 min. Subsequently, body bends were counted for 30 s. A single body bend is defined as one sinusoidal movement of a worm. In the locomotion assay, only live worms were counted for the assays. Approximately 20 worms were examined in each experiment.

### *C. elegans* memory assays

Chemotaxis to water-soluble compounds was performed at 20° C, on 9 cm agar plates as described previously [39–42]. The chemotaxis index was calculated by subtracting the number of animals found at the trap from the number of animals at the source of the chemical, divided by the total number of animals subjected to the assay [39]. The resulting values were expressed as percentiles. Three distinct populations of 100 adults (for each strain) were scored during the assay period. For all experiments, spermidine (5 mM) nematodes were treated from eggs. Both naive and conditioned animals were challenged for 1h with gradients of 100mM NaCl. For conditioning, animals were exposed to 20mM NaCl on agar plates devoid of bacterial food, for 4h, prior to assaying chemotaxis to the respective compound. Approximately 40 worms were examined in each experiment, three technical replicates were performed for all nematode experiments.

### Quantitative RT-PCR

Quantitative RT-PCR was performed on nematodes which were cultured and treated as described above. Total RNA was isolated using TRIzol (Invitrogen, USA) following the manufacture’s protocol. Then, total RNA was converted into complementary DNA (cDNA) using PrimeScript^TM^ RT Reagent Kit (Takara, Japan) for RT–qPCR (Bio-Rad, CA, USA). cDNA was diluted to a ratio of 1:10 with TB Green® Premix Ex Taq™ (Takara, Japan) with ROX qRT-PCR (Bio-Rad, CA, USA), forward and reverse primers of specific genes. The qPCR primers for *bec-1* were 5’- ATG GAT GCT CAA GTG GCG ACA C-3’ (forward primer, F) and 5’- TCC AGC TCC TTC TCC TCA TCG G-3’ (reverse primer, R), for *sqst-1* were 5’-TCC CTC CAT CTC CAA CCA TCG G-3’(F) and 5’- GCT GCA CGG GTC TCC TCA TTG-3’®. for *pink-1* were 5’- ACG CTT CCT GCC GAG AAT ATT TCC -3’(F) and 5’-CGA CCG TGG CGA GTT ACA AGG-3’®, for *pdr-1* were 5’-CGG CGG TCT GCG AAG AAT GC-3’(F) and 5’- TCC TTC CCA TCA CAA ACA CAG CAC-3’®The actin (*act-1*) with primers 5'-CGC CAT CCT CCG TCT TGA CTT G-3' (F) and 5'- GCT CAG CGG TGG TGG TGA AAG-3' ®) was used as an internal control.

### RNA interference knockdown

RNAi experiments were carried out by feeding worms with bacteria expressing RNA against the gene of interest (or control bacteria carrying the empty L4440 vector). RNAi clones (transformed bacteria) were grown at 37° C overnight and then seeded onto NGM plates containing ampicillin (100 μg/ml). Expression of RNA was induced by the addition of 1 mM IPTG before eggs were added to the bacterial lawns. RNAi clones were obtained from Ahringer’s RNAi library [40].

### Statistical analysis

All values are expressed as mean ± SD. A two-tailed Student’s t-test was used to compare means between two groups. Statistical analysis was performed by using GraphPad Prism 8.0 (GraphPadPrism Software, La Jolla, CA).

## Supplementary Material

Supplementary Figures
