# Supplementary material for: Spermidine inhibits neurodegeneration and delays aging via the PINK1-PDR1-dependent mitophagy pathway in C. elegans
Source: Aging (Albany NY). 2020 Sep 9;12(17):16852–66. doi: 10.18632/aging.103578 (PMC7521492; doi:10.18632/aging.103578)
Supplement: Supplementary Figures [file aging-12-103578-s001..pdf]

SUPPLEMENTARY FIGURES

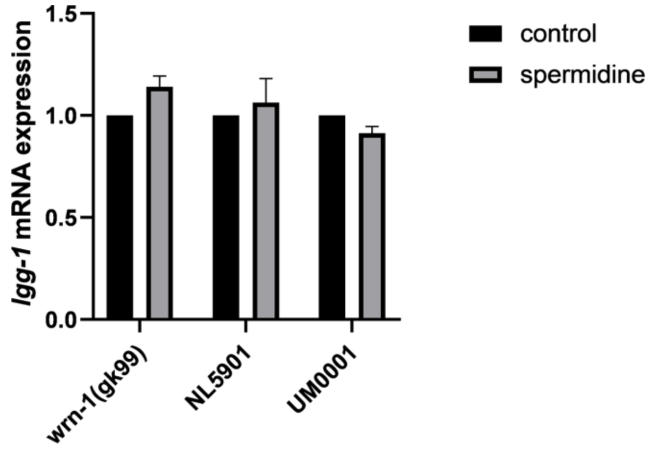

Supplementary Figure 1. Effect of 5mM spermidine treatment on autophagy-related gene *lgg-1* of *wrn-1(gk99)*, NL5901, UM0001 worms.

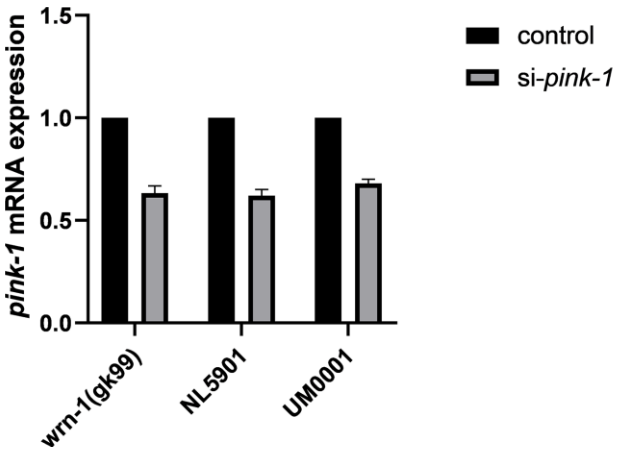

Supplementary Figure 2. RNAi knockdown of *pink-1* in *wrn-1(gk99)*, NL5901, UM0001 worms.

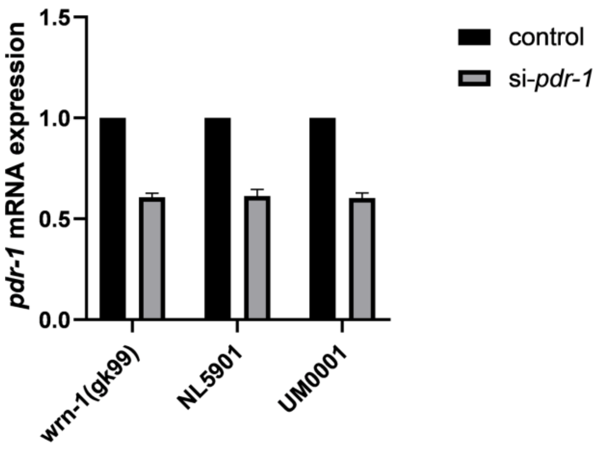

Supplementary Figure 3. RNAi knockdown of *pdr-1* in *wrn-1(gk99)*, NL5901, UM0001 worms.
